# Intrabladder pressure as predictor of intra-abdominal pressure in horses

**DOI:** 10.1371/journal.pone.0223705

**Published:** 2019-10-10

**Authors:** Vanessa B. de Paula, Paulo A. Canola, Gabriela G. Rivera, Dárcio Z. Filho, Gabriel P. D. Amaral, Guilherme C. Ferraz, Antônio S. Ferraudo, Júlio C. Canola

**Affiliations:** 1 Graduate Program in Veterinary Surgery, São Paulo State University (Unesp), School of Agricultural and Veterinarian Sciences, Jaboticabal, SP, Brazil; 2 Department of Veterinary Medicine and Surgery, São Paulo State University (Unesp), School of Agricultural and Veterinarian Sciences, Jaboticabal, SP, Brazil; 3 Residency Program in Large Animal Surgery, São Paulo State University (Unesp), School of Agricultural and Veterinarian Sciences, Jaboticabal, SP, Brazil; 4 Department of Animal Morphology and Physiology, São Paulo State University (Unesp), School of Agricultural and Veterinarian Sciences, Jaboticabal, SP, Brazil; 5 Department of Exact Sciences, São Paulo State University (Unesp), School of Agricultural and Veterinarian Sciences, Jaboticabal, SP, Brazil; University College Dublin, School of Veterinary Medicine, IRELAND

## Abstract

**Objectives:**

To investigate effects of postural changes and bladder distention on intrabladder pressure whilst estimating intra-abdominal pressure in horses.

**Design:**

Two-year cohort study. Patients admitted for elective surgical procedures unrelated to gastrointestinal or genitourinary tract.

**Setting:**

School of Agricultural and Veterinarian Sciences, Jaboticabal, SP, Brazil.

**Animals:**

20 adult horses, 11 males (stallions and geldings) and 9 females; between 3.5 and 12 years, weighing 350 to 500 kg.

**Interventions:**

Intra-abdominal pressure was directly-recorded through abdominocentesis at the ventral midline with a fluid-filled system. Intrabladder pressure was obtained from a bladder catheter with the fluid-filled system zeroed at the level of the tuber ischia with patients in dorsal recumbency or pubic symphysis if in lateral recumbency.

**Measurements and main results:**

Body position directly influenced intra-abdominal pressure. In dorsal recumbency, intra-abdominal pressure differed (p < 0.05) from intrabladder pressure at end-inspiration and end-expiration regardless of whether the bladder was empty or distended. There was no correlation nor association between the two pressures in this body position. In lateral recumbency a difference (p <0.05) between intra-abdominal pressure and intrabladder pressure was recorded at end-inspiration with the bladder distended with 25 ml, and at end-expiration for distension volumes of 25 ml and 50 ml. There was a strong correlation between both pressures for left and right lateral recumbency, regardless of the distension volume. Ordinary least product (OLP) regression analysis showed no fixed or proportional bias between both pressures for distension volume of 50 ml, at both end-inspiration and end-expiration.

**Conclusions:**

Indirect assessment of equine intra-abdominal pressure cannot be made in dorsal recumbency. For that purpose, patients should be in left lateral recumbency with the bladder distended with 50 ml. Values can be recorded at end-inspiration or end-expiration.

**Restriction:**

Occlusion of the catheter tip by the bladder wall when minimally distended.

## Introduction

Intra-abdominal pressure (IAP) is defined as the pressure within the abdominal cavity resulting from the interaction between the abdominal wall and abdominal contents [1,2]. In people, the abdomen is considered primarily fluid in character and behaves as a homogeneous hydraulic fluid system, conforming to Pascal’s law. It follows that the pressure exerted on any part of the abdominal cavity will be distributed equally throughout it, being isotropic when the fluid points are at the same absolute height. This allows IAP to be estimated by the pressure exerted in hollow organs, such as the bladder [1,3–6], rectum or stomach [7,8].

Several clinical and therapeutic conditions have been associated with the increase in IAP in men [1,6]. It is common knowledge that the increase in IAP can potentially cause detrimental effects in the body with the manifestations of intra-abdominal hypertension (IAH). The point to which multiple organ disfunction syndrome (MODS) manifests as a result of severe IAH is termed abdominal compartment syndrome (ACS) [1,2,4–6]. Both conditions are highly detrimental and potentially fatal [1,2,4,5], and can only be diagnosed by measuring IAP [4]. In so, they have been extensively studied in men, but still poorly investigated in the horse [2,6].

The gold standard method for indirect assessment of IAP in man, recognized by the World Society for Abdominal Compartment Syndrome (WSACS), is intrabladder pressure (IBP) recordings obtained through a fluid filled manometry system. For this, the patient must be in supine position (i.e. dorsal recumbency) [1,6,9,10]. Bladder distension with small volumes of saline solution is necessary for optimal readings [11].

Originally, larger volumes of saline were used for bladder distension (more than 100 ml), but it was discovered that such volumes directly influence IBP values [12]. This finding has also been reported [13] in horses. Currently, WSACS recommends that the bladder of children up to seven years of age should be distended with 1.0 ml / kg of saline solution with a maximum volume of 25 ml in young people / adults [10,11,14]. Furthermore, IBP readings should be performed at end-expiration [4,10,11,14,15].

Previous attempts to estimate IAP by intragastric pressure (IGP) [16] or IBP measurements in horses using methodologies previously established in man were unsuccessful [17,18]. More recently, methodological variations in IGP [19] and IBP recordings [13] were also attempted in horses with unfavorable results. In all of these studies the animals were standing during measurement of IBP.

Due to the failure of these previous attempts to indirectly estimate IAP in horses, it was suggested that the equine abdomen does not conform to Pascal's law [6,20]. Since then, IAP has been directly measured in horses. Direct assessment of IAP has been proven to be a reliable method for IAP readings in horses [6], but is also susceptible to reading errors attributed to failure to position the manometer at the reference point, spontaneous oscillations that can distort the IAP curve, presence of air bubbles in the circuit and inadequate calibration of the equipment [4].

Therefore, we sought to correlate intra-abdominal pressure with intrabladder pressure in horses, and validate the indirect method of IAP acquisition through IBP. In contrast to previous attempts, we considered whether variations in the subjects' body position and the volume of bladder distension were factors that affect the efficacy of the method, as they do in man.

## Materials and methods

### Animals

The study was approved and supervised by the institutional animal care and use committee of the São Paulo State University (Unesp), School of Agricultural and Veterinarian Sciences, Jaboticabal, SP, Brazil (CEUA protocol # 858/16). A total of 20 horses, 11 males (stallions and geldings) and 9 females, between 3.5 and 12 years of age and weighing 350 to 500 kg were included in the study. Subjects were divided into two groups according to their body position during elective surgical procedure: lateral recumbency (n = 10) and dorsal recumbency (n = 10). All patients were admitted to the hospital’s large animal surgery service for elective surgeries not related to the gastrointestinal or genitourinary tract. Prior to the clinical trials and the surgical procedure, all patients underwent clinical and hematological evaluation. The study was carried out with the consent of the owners.

### Patient preparation

Food was withheld for 12 hours and water for one hour prior to surgery. Patients were pre-medicated with xylazine hydrochloride 0.5–1.0 mg /kg, given intravenously. After five minutes, ketamine hydrochloride 2.0 mg /kg in combination with midazolam maleate 0.1 mg /kg, was administered intravenously. Following anesthetic induction and orotracheal intubation, patients were hoisted and positioned on the surgical table in either dorsal or lateral recumbency, depending on the surgical procedure to be performed. Subsequently, the orotracheal tube was connected to the Mallard® inhalation anesthetic device previously saturated with isoflurane, for anesthetic maintenance (1.5 to 2.2 CAM). The anesthetic equipment was set for pressure-controlled mechanical ventilation (20 to 25 mmHg) with inspired fraction of oxygen (FiO_2_) of 1.0 and inspiratory/expiration ratio of 1: 3.

### Instrumentation for IAP registration

For direct IAP acquisition, the abdominal cavity was assessed at the linea alba, approximately 10 cm caudal to the xiphoid [6]. Initially, a 5.0 x 5.0 cm area of hair coat was shaved at the predetermined landmark, surgically prepared with 2% chlorhexidine gluconate and 70% ethyl alcohol and anesthetically blocked with 2.0 ml of lidocaine hydrochloride 2%. Then, a small stab incision through the skin and subcutaneous tissue, and partially through the linea alba, was made to allow the insertion of a 7.5-cm long teat cannula into the abdominal cavity. Its distal extremity was coupled to a sealed water manometry system, pre-filled with 0.9% sterile saline solution, through a three-way stopcock. The manometry system was zeroed at the level of the cannula and IAP was recorded at end-inspiration and end-expiration for ten consecutive respiratory cycles.

### Instrumentation for IBP registration

**Fig 1:**
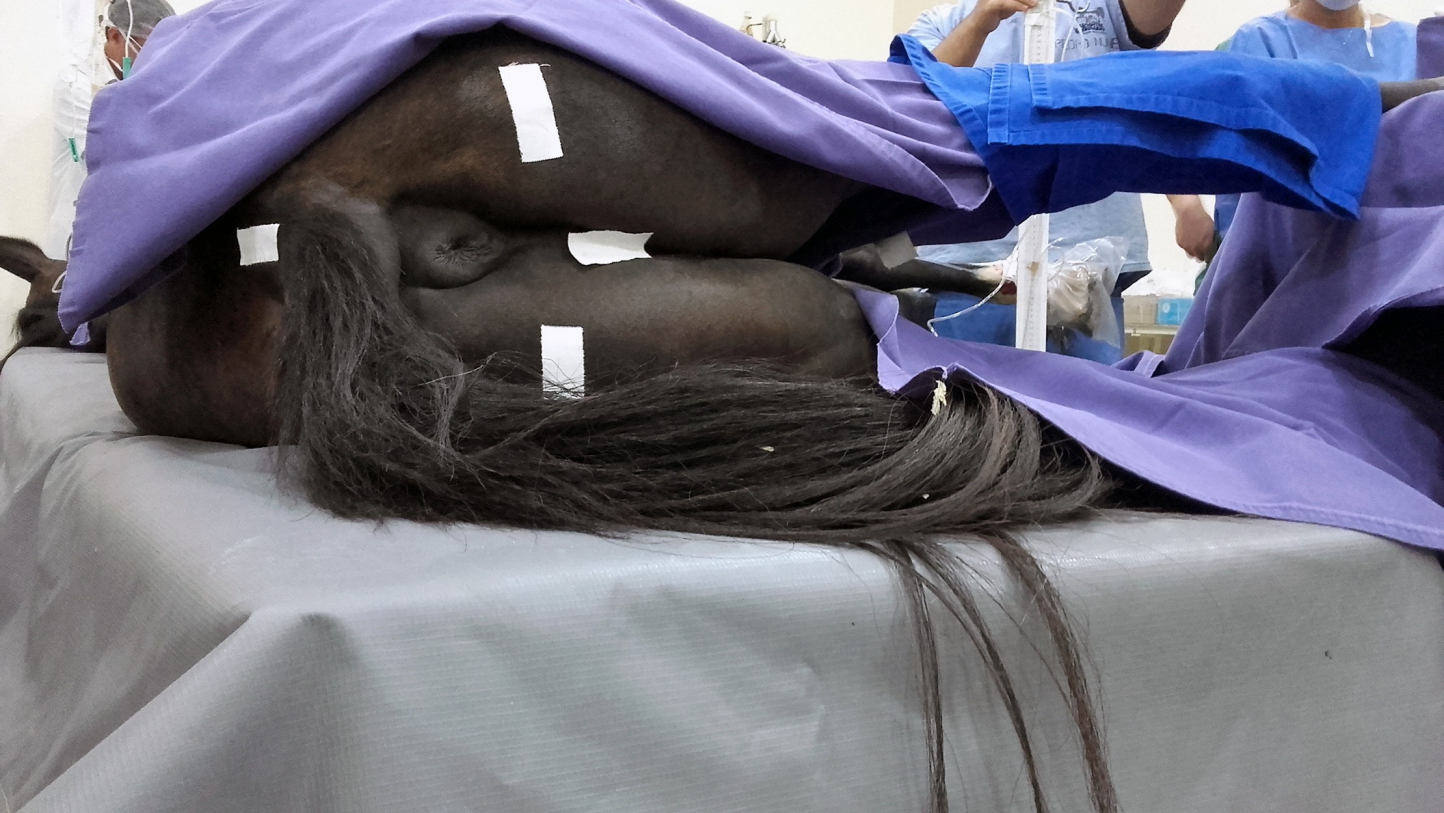
Intrabladder pressure monitoring with the patient in left lateral recumbency. Intrabladder pressure monitoring with the patient in left lateral recumbency. Water manometry system was zeroed at the level of the pubic symphysis in each patient.

The patients’ bladder was catheterized for IBP recordings (Fig 1). A Foley’s catheter of appropriate diameter was used in female patients’ and a Levine’s gastric tube in the males. The external extremity of the catheters was then coupled with a system comprised of three three-way stopcocks connected in line, attached to the sealed water manometry system pre-filled with 0.9% NaCl saline solution, as described elsewhere [21]. The manometry system was zeroed at the level of the tuber ischia in patients in dorsal recumbency or at the level of the pubic symphysis in those in lateral recumbency [13,18].

### Experimental procedure

Then bladder was emptied (IBP0ml), then a five-minute adaptation period was given before recording IBP and following each bladder distension with the predetermined volumes of 25 ml (IBP25ml), 50ml (IBP50ml) and 100 ml (IBP100ml). This was to allow pressure to reach equilibrium within the bladder and to minimize the effects of the detrusor muscle on IBP values during recording [4,13,18,22]. Intrabladder pressure values were then recorded simultaneously and in the same way as IAP recordings. Both IAP and IBP values were initially obtained in cm H_2_O and subsequently converted to mmHg (1.0 cm H2O = 0.73556 mmHg). System viability during both pressure recordings was ascertain by observation of oscillatory movements of the water column in synchrony with the respiratory cycle.

Following IBP recordings with the bladder emptied (IBP_0ml_) it was checked for residual volumes. Once it was determined that the bladder was empty (i.e. absence of urine or saline on aspiration) it was distended with 25 ml of 0.9% NaCl solution. Following the five-minute adaptation period, IBP was recorded again. The bladder was then emptied again and distended with 50 ml. The procedure was repeated and IBP was also recorded for all distension volumes. Intra-abdominal pressure was also recorded for each bladder distension volume.

Once both pressure recordings were completed, bladder contents were aspirated and the Foley or Levine catheter removed. If the surgical procedure was complete the patient was moved to the anesthetic recovery room.

### Statistical methods

Data were initially subjected to the Shapiro-Wilk normality test. Kruskal-Wallis’ followed by post-hoc Dunnett’s test was then used to detect differences between IAP and IBP values. The post-hoc Student-Newman-Keuls’ test was used to evaluate the influence of bladder distension volume on IBP. The correlation between both pressures was verified by Spearman’s test and the agreement between both pressures by the Bland-Altman’s method. The analyses were performed using SigmaPlot software version 12.0—Systat Software Inc. Secondly, the ordinary least products regression analysis (OLP) was used to identify fixed bias by means of a 95% confidence interval (CI) of the y-interceptor. If zero is included in the interceptor IC, there is no fixed bias. The proportional bias was determined from the 95% CI for slope. In this case, if the CI includes the value of 1.0 there is no proportional bias [23,24]. The R program (version 3.2.5)–Free Software Foundation Inc. was used to assess this. Data were also submitted to exploratory multivariate analysis of multiple correspondence to verify association between variables. For this, the StatSoft 7 software—Dell Inc. was used. The significance of all tests was set at p ≤ 0.05.

## Results

**Fig 2:**
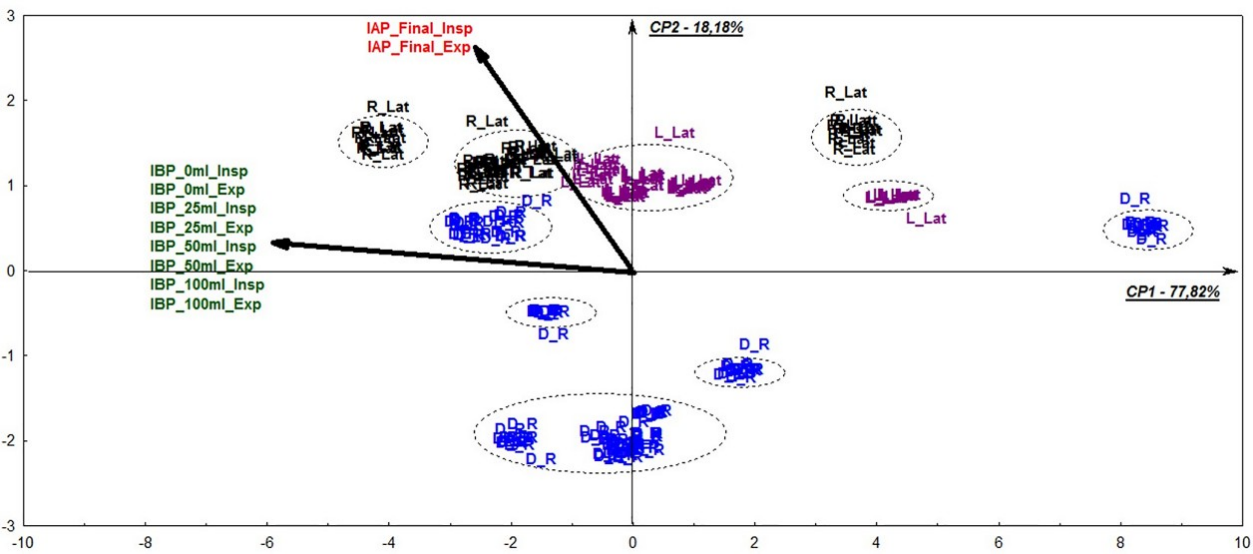
Exploratory multivariate analysis of multiple correspondence of 20 equine patients anesthetized in different body positions. Perceptual multiple correspondence (multivariate exploratory) analysis map of 20 horses that had IAP and IBP recorded in different body positions during elective surgical procedures. The present data express 96% of the data variability (Factor 1 + Factor 2). Patients remained in either dorsal recumbency (D_R) or right (R_Lat) or left (L_Lat) lateral recumbency. Intra-abdominal pressure was recorded at end-inspiration (IAP_Final_Insp) and end-expiration (IAP_Final_Exp). Intrabladder pressure was obtained simultaneously with IAP, with the bladder empty (IBP_0ml_Insp; IBP_0ml_Exp) and distended with 25 ml (IBP_25ml_Insp; IBP_25ml_Exp), 50 ml (IBP_50ml_Insp; IBP_50ml_Exp) and 100 ml (IBP_100ml_Insp; IBP_100ml_Exp) of 0.9% NaCl solution.

Body position directly influenced IAP readings (Table 1). This observation was verified in both univariate and multivariate statistical analysis. In the latter, both pressures constituted distinct processes, with no association between them, independent of whether or not the bladder was distended (Fig 2). However, there was a significant correlation between IAP and either right or left lateral recumbency, for both phases of the respiratory cycle. Data related to lateral recumbency were more evenly distributed, especially for the left side. Data from the dorsal recumbency was considerably more dispersed and poorly associated with IAP (Fig 2). The strongest correlation between IAP and IBP was found to be at end-inspiration (rs = 0.931, p < 0.001) and end-expiration (rs = 0.937, p < 0.001) in patients kept in left lateral recumbency.

**Table 1:** Intra-abdominal pressure obtained in 20 equine patients in dorsal recumbency (n = 10) and right (n = 5) and left (n = 5) lateral recumbency.

Patients	IAP D.R.		Patients	IAP R.R.		IAP L.R.	
	E.I.	E. E.		E.I.	E. E.	E.I.	E. E.
**1**	-3.678	-4.340	**1**			9.930	8.753
**2**	2.501	2.574	**2**			8.312	6.877
**3**	-6.62	-5.884	**3**			4.046	3.678
**4**	-0.956	-2.611	**4**			8.496	7.981
**5**	-6.105	-7.025	**5**			7.521	6.583
**6**	8.349	7.760	**6**	8.680	6.841		
**7**	7.76	7.135	**7**	11.328	10.298		
**8**	-4.781	-5.149	**8**	12.026	9.415		
**9**	-6.215	-6.914	**9**	11.732	10.666		
**10**	-4.266	-5.039	**10**	16.256	12.817		
$\bar{X}$ **± SD**	**-3.678 ± 5.7**	**-4.413 ± 5.6**	$\bar{X}$ **± SD**	**11.769 ± 3.2**	**10.298± 2.3**	**8.091 ± 2.7**	**6.436 ± 2.6**

Pressure values expressed in mmHg.

Mean ($\bar{X}$) ± standard deviation (SD) of intra-abdominal pressures (IAP) obtained at end-inspiration (EI) and end-expiration (EE) in 10 patients in dorsal recumbency (IAP D.R.) and 10 patients in lateral recumbency [five in right lateral recumbency (IAP R.R.) and five in left lateral recumbency (IAP L.R.)], during inhalation anesthesia with mechanical ventilation for completion of elective surgical procedures.

As previously mentioned, intra-abdominal pressure varied (p <0.001) in response to patient body position on univariate statistical analysis. There was no correlation between recorded IAP in dorsal vs lateral recumbency at either end-inspiration (rs = 0.127, p = 0.209) or end-expiration (rs = 0.0393, p = 0.697). When lateral recumbency results were categorized into right and left, IAP values in dorsal recumbency moderately correlated with those obtained in patients in left lateral recumbency at both end-inspiration (rs = 0.622, p <0.001) and end-expiration (rs = 0.430, p <0.001). Interestingly, IAP values in dorsal recumbency had a weak, and inverse, correlation with values recorded in right lateral recumbency at end-inspiration (rs = -0.402, p <0.001).

The IAP and IBP obtained with patients in dorsal recumbency are expressed in Table 2. There was a difference (p <0.05) and low agreement between both pressures at end-inspiration and end-expiration (Table 3), regardless of whether IBP had been obtained with an emptied or distended bladder. There was an insignificant correlation between IAP and IBP obtained at end-inspiration with the distended bladder, regardless of the volume used for distension: IBP_25ml_ (rs = 0.293, p <0.00318); IBP_50ml_ (rs = 0.329, p <0.000163); and IBP_100ml_ (rs = 0.370, p <0.000163). A weak correlation was observed between IAP and IBP_0ml_ (rs = 0.214, p = 0.0326) and IBP_25ml_ (rs = 0.319, p = 0.00126) at end-expiration. For both IBP_50ml_ (rs = 0.425, p <0.001) and IBP_100ml_ (rs = 0.434, p <0.001) a moderate correlation was observed, with fixed bias, but not proportional bias, between both on ordinary least products regression analysis (OLP) (Table 4).

**Table 2:** Intra-abdominal pressure and intrabladder pressure obtained from 10 equine patients in dorsal recumbency during elective surgical procedures.

Patients	IAP		IBP							
	E. I.	E. E.	0 ml		25 ml		50 ml		100 ml	
			E. I.	E. E.	E. I.	E. E.	E. I.	E. E.	E. I.	E. E.
**1**	-3.678	-4.34	6.62	5.112	7.061	5.517	6.767	5.443	7.282	5.627
**2**	2.501	2.574	6.62	5.112	7.061	5.517	6.767	5.443	7.282	5.627
**3**	-6.62	-5.884	9.93	8.349	9.489	7.135	10.151	8.606	10.886	8.9
**4**	-0.956	-2.611	-1.508	-2.979	-3.567	-4.193	-2.427	-3.494	-2.979	-3.715
**5**	-6.105	-7.025	9.599	9.084	9.562	8.827	9.82	9.084	7.613	6.877
**6**	8.349	7.76	10.96	10.555	11.217	10.85	11.916	11.291	13.939	13.498
**7**	7.76	7.135	11.512	10.371	10.187	8.827	11.291	9.93	13.314	11.033
**8**	-4.781	-5.149	7.356	6.62	9.011	7.76	9.231	8.054	9.599	8.422
**9**	-6.215	-6.914	8.716	7.687	8.827	7.723	9.121	7.797	9.194	8.091
**10**	-4.266	-5.039	10.187	9.415	12.174	9.856	12.431	9.856	14.822	12.026
$\bar{X}$ **± SD**	**-3.97 ± 5.7**	**-4.69 ± 5.7**	9.16 ± 3.8*	8.02 ± 3.9*	9.25 ± 4.4*	7.74 ± 4.2*	9.53 ± 4.3*	8.33 ± 4.2*	9.40 ± 5.1*	8.26 ± 4.8*

Pressure values expressed in mmHg.

*Statistically different from IAP (Dunnett's test, p ≤ 0.05).

Mean ($\bar{X}$) and standard deviation (SD) of intra-abdominal pressure (IAP) and intrabladder pressure (IBP) of 10 horses in dorsal recumbency during elective surgical procedures. Both pressures were recorded at end-inspiration (E.I.) and end-expiration (E.E.). Intrabladder pressure was recorded with an emptied bladder (0ml) and when distended with predetermined volumes of 25ml, 50ml and 100ml.

**Table 3:** Bland-Altman limits of agreement method between intra-abdominal pressure and intrabladder pressure of 20 equine patients in dorsal recumbency (n = 10) or lateral recumbency (n = 10) during elective surgical procedures.

**Distension Volume**	Dorsal Recumbency						Lateral Recumbency					
	E. I.			E. E.			E. I.			E. E.		
	B	S. D.	CI 95%	B	S. D.	CI 95%	B	S. D.	CI 95%	B	S. D.	CI 95%
**0 ml**	9.5	6.0	8.3–10.7	9.1	5.7	8.0–10.3	-1.0	2.1	-1.4 - -0.6	-0.9	1.7	-1.3 - -0.6
**25 ml**	10.0	6.5	8.6–11.2	9.3	6.0	8.1–10.5	-1.3	1.5	-1.6 - -1.0	-1.4	1.4	-1.7 - -1.1
**50 ml**	10.3	6.3	9.1–11.6	9.7	5.8	8.5–10.8	**-0.9**	1.8	-1.3 - -0.5	-2.6	1.8	-3.0 - -2.3
**100 ml**	11.0	6.2	9.8–12.2	10.2	5.6	9.1–11.3	**-0.1**	2.1	-0.5–0.3	-1.8	1.8	-2.2 - -1.5

Pressure values expressed in mmHg.

Bias (B), standard deviation (SD) and confidence interval (CI) between the mean values of intra-abdominal pressure (IAP) and intrabladder pressure (IBP), obtained by the Bland-Altman limits of agreement method. The IAP and IBP values of 20 equine patients were obtained at end-inspiration (EI) and end-expiration (EE) whilst in dorsal (n = 10) or lateral recumbency (n = 10). Intrabladder pressure was recorded with the bladder emptied (0 ml) and subsequently distended with predetermined volumes of 25ml, 50ml and 100ml 25 ml, 50 ml and 100 ml.

**Table 4:** Ordinary least product regression analysis (OLP) between intra-abdominal pressure and intrabladder pressure of 20 equine patients in dorsal (n = 10) or lateral recumbency (n = 10) during elective surgical procedures.

Proportional	*n*	*r*	*a*	95% CI	*b*	95% CI	PB	CB
IAP*di*-IBP*di*0	10	0.10	8.33	-0.74–2.30	0.78	6.82–9.85	No	Yes
IAP*di*-IBP*di*25	10	0.07	8.85	-0.73–2.55	0.91	7.21–10.49	No	Yes
IAP*di*-IBP*di*50	10	0.10	9.26	-0.73–2.50	0.89	7.64–10.87	No	Yes
IAP*di*-IBP*di*100	10	0.22	10.03	-0.70–2.76	1.03	8.30–11.76	No	Yes
IAP*de*-IBP*de*0	10	0.18	7.93	-0.73–2.31	0.79	6.41–9,45	No	Yes
IAP*de*-IBP*de*25	10	0.19	8.18	-0.72–2.45	0.87	6.59–9.76	No	Yes
IAP*de*-IBP*de*50	10	0.20	8.57	-0.72–2.43	0.86	6.99–10.15	No	Yes
IAP*de*-IBP*de*100	10	0.30	9.23	-0.68–2.61	0.96	7.59–10.88	No	Yes
IAP*lli*-IBP*lli*0	5	0.85	-4.29	-0.01–3.08	1.53	-5.83 - -2.74	No	Yes
IAP*lli*-IBP*lli*25	5	0.95	-3.73	0.26–2.48	1.37	-4.85 - -2.62	No	Yes
**IAP*lli*-IBP*lli*50**	**5**	**0.91**	**-0.84**	**-0.09–2.11**	**1.01**	**-1.94–0.26**	**No**	**No**
IAP*lli*-IBP*lli*100	5	0.94	-3.41	0.21–2.65	1.43	-4.64 - -2.19	No	Yes
IAP*lle*-IBP*lle*0	5	0.92	-3.41	0.06–2.80	1.43	-4.78 - -2.04	No	Yes
IAP*lle*-IBP*lle*25	5	0.99	-2.41	0.52–1.87	1.20	-3.08 - -1.74	No	Yes
**IAP*lle*-IBP*lle*50**	**5**	**0.99**	**0.20**	**0.24–1.71**	**0.98**	**-0.54–0.93**	**No**	**No**
IAP*lle*-IBP*lle*100	5	0.99	-2.52	0.53–2.17	1.35	-3.33 - -1.70	No	Yes
IAP*lri*-PIV*lri*0	5	0.67	-3.69	-0.73–3.04	1.15	-5.57 - -1.80	No	Yes
IAP*lri*-IBP*lri*25	5	0.89	-8.20	-0.16–3.25	1.55	-9.90 - -6.49	No	Yes
IAP*lri*-IBP*lri*50	5	0.90	-9.53	-0.07–3.37	1.65	-11.25 - -7.81	No	Yes
IAP*lri*-IBP*lri1*00	5	0.86	-10.16	-0.15–3.78	1.82	-12.12 - -8.19	No	Yes
IAP*lre*-IBP*lre*0	5	0.80	-5.37	-0.27–3.11	1.42	-7.06 - -3.68	No	Yes
IAP*lre*-IBP*lre*25	5	0.95	-9.18	0.38–3.14	1.76	-10.57 - -7.80	No	Yes
IAP*lre*-IBP*lre*50	5	0.93	-10.28	0.35–3.38	1.86	-11.80 - -8.76	No	Yes
IAP*lre*-IBP*lre*100	5	0.94	-11.37	0.55–3.67	2.11	-12.94 - -9.81	No	Yes

r, product–moment correlation coefficient a and b—coefficients in the ordinary least products regression model E(A) = a+b(B)22; a, LT (y axis) intercept; and b, slope. PB, proportional bias; CB constant bias *p<0.0001. Intra-abdominal pressure in dorsal recumbency at end-inspiration (IAP*di*), intrabladder pressure in dorsal recumbency at end-inspiration with emptied bladder (IBP*di*0), distended with 25ml (IBP*di*25), 50ml (IBP*di*50), and 100ml (IBP*di*100). Intra-abdominal pressure in dorsal recumbency at end-expiration (IAP*de*), intrabladder pressure in dorsal recumbency *a*t end-expiration with an emptied bladder (IBP*de*0), distended with 25ml (IBP*de*25), 50ml (IBP*de*50), and 100ml (IBP*de*100). Intra-abdominal pressure in left lateral recumbency at end-inspiration (IAP*lli*), intrabladder pressure in left lateral recumbency at end-inspiration with emptied bladder (IBP*lli0*), distended with 25ml (IBP*lli25*), 50ml (IBP*lli*50), and 100ml (IBP*lli*100). Intra-abdominal pressure in left lateral recumbency at end-expiration (IAP*lle*), intrabladder pressure in left lateral recumbency at end-expiration with emptied bladder (IBP*lle*0), distended with 25ml (IBP*lle*25), 50ml (IBP*lle*50), and 100ml (IBP*lle*100). Intra-abdominal pressure in right lateral recumbency at end-inspiration (IAP*lri*), intrabladder pressure in right lateral recumbency at end-inspiration with emptied bladder (IBP*lri*0), distended with 25ml (IBP*lri*25), 50ml (IBP*lri*50), and 100ml (IBP*lri*100). Intra-abdominal pressure in right lateral recumbency at end-expiration (IAP*lre*), intrabladder pressure in right lateral recumbency at end-expiration with emptied bladder (IBP*lre*0), distended with 25ml (IBP*lre*25), 50ml (IBP*lre*50), and 100ml (IBP*lre*100).

When assessing the influence of distension volume on IBP values in patients in dorsal recumbency, IBP_0ml_ differed (p <0.05) from IBP_25ml_, both at end-inspiration and end-expiration. Correlation coefficient (rs) varied between 0.697 (IBP_0ml_ and IBP_25ml_) and 0.922 (IBP_50ml_ and IBP_100ml_) at end-inspiration, and from 0.689 (IBP_0ml_ and IBP_25ml_) to 0.936 (IBP_25ml_ and IBP_100ml_) at end-expiration (p <0.001). However, there was strong correlation between IBP recorded with the emptied bladder and whilst distended, regardless of the volume used for bladder distension.

Intra-abdominal pressure and IBP of patients in lateral recumbency are shown in Table 5. At end-inspiration, there was a difference (p <0.05) between IAP and IBP_25ml_. At end-expiration, both pressures differed for IBP_25ml_ and IBP_50ml_. The correlation between IAP and IBP remained very strong, regardless of the distension volume. At end-inspiration, the coefficient of correlation (rs) between IAP and IBP varied from 0.839 for the IBP_0ml_ to 0.919 for the IBP_25ml_. At end-expiration, the correlation coefficient (rs) varied from 0.827 for the IBP_0ml_ to 0.936 for the IBP_25ml_. Unlike in the supine position, the Bland-Altman showed agreement between both pressures for patients in lateral recumbency, with the most significant results observed at end-inspiration for IBP_50ml_ and IBP_100ml_ (Table 3).

**Table 5:** Intra-abdominal pressure and intrabladder pressure of 10 equine patients in lateral recumbency during elective surgical procedures.

Patients	IAP		IBP							
	E. I.	E. E.	0 ml		25 ml		50 ml		100 ml	
			E. I.	E. E.	E. I.	E. E.	E. I.	E. E.	E. I.	E. E.
**1**	9.930	8.753	10.224	7.797	9.121	7.356	10.298	7.208	10.298	7.650
**2**	8.312	6.877	9.562	8.827	7.981	6.988	8.091	7.356	8.900	7.356
**3**	4.046	3.678	2.280	1.067	2.317	0.933	4.413	2.942	2.795	1.471
**4**	8.496	7.981	6.988	6.620	7.760	6.988	8.385	7.356	9.562	8.459
**5**	7.521	6.583	8.569	6.399	6.455	5.480	7.135	5.278	7.466	6.510
**6**	8.680	6.841	4.671	3.420	3.347	2.096	2.905	1.839	3.273	2.207
**7**	11.328	10.298	10.224	9.158	10.702	8.532	9.783	7.797	13.681	11.033
**8**	12.026	9.415	12.505	10.482	11.512	9.599	11.806	9.893	12.836	10.960
**9**	11.732	10.666	11.843	10.886	11.585	9.415	11.659	9.562	11.916	9.673
**10**	16.256	12.817	11.475	10.114	14.711	12.505	14.969	12.725	16.532	14.656
$\bar{X}$ **± SD**	**9.31 ± 3.3**	**8.37 ± 2.6**	**9.89 ± 3.3**	**8.31 ± 3.2**	8.55 ± 3.8*	7.17 ± 3.5*	**9.08 ± 3.6**	7.40 ± 3.2*	**9.93 ± 4.4**	**8.05 ± 4.0**

Pressure values expressed in mmHg.

* Statistically different from IAP (Dunnett's test, p ≤ 0.05).

Mean ($\bar{X}$) and standard deviation (SD) of intra-abdominal pressure (IAP) and intrabladder pressure (IBP) of 10 equine patients in lateral recumbency during elective surgical procedures. Both pressures were recorded at end-inspiration (E.I.) and end-expiration (E.E.). Intrabladder pressure was recorded with the bladder emptied (0ml) and distended with predetermined volumes of 25ml, 50ml and 100ml.

As observed with the dorsal recumbency, distension volumes also directly affected IBP values of patients in lateral recumbency (p = 0.055). However, there was also a strong correlation between IBP recorded with an emptied or distended bladder, regardless of the distension volume. The correlation coefficient (rs) varied from 0.828 (IBP_0ml_ and IBP_100ml_) to 0.955 (IBP_25ml_ and IBP_50ml_) at end-inspiration, and from 0.836 (IBP_0ml_ and IBP_100ml_) to 0.951 (IBP_25ml_ and IBP_50ml_) at end-expiration (p <0.001).

**Fig 3:**
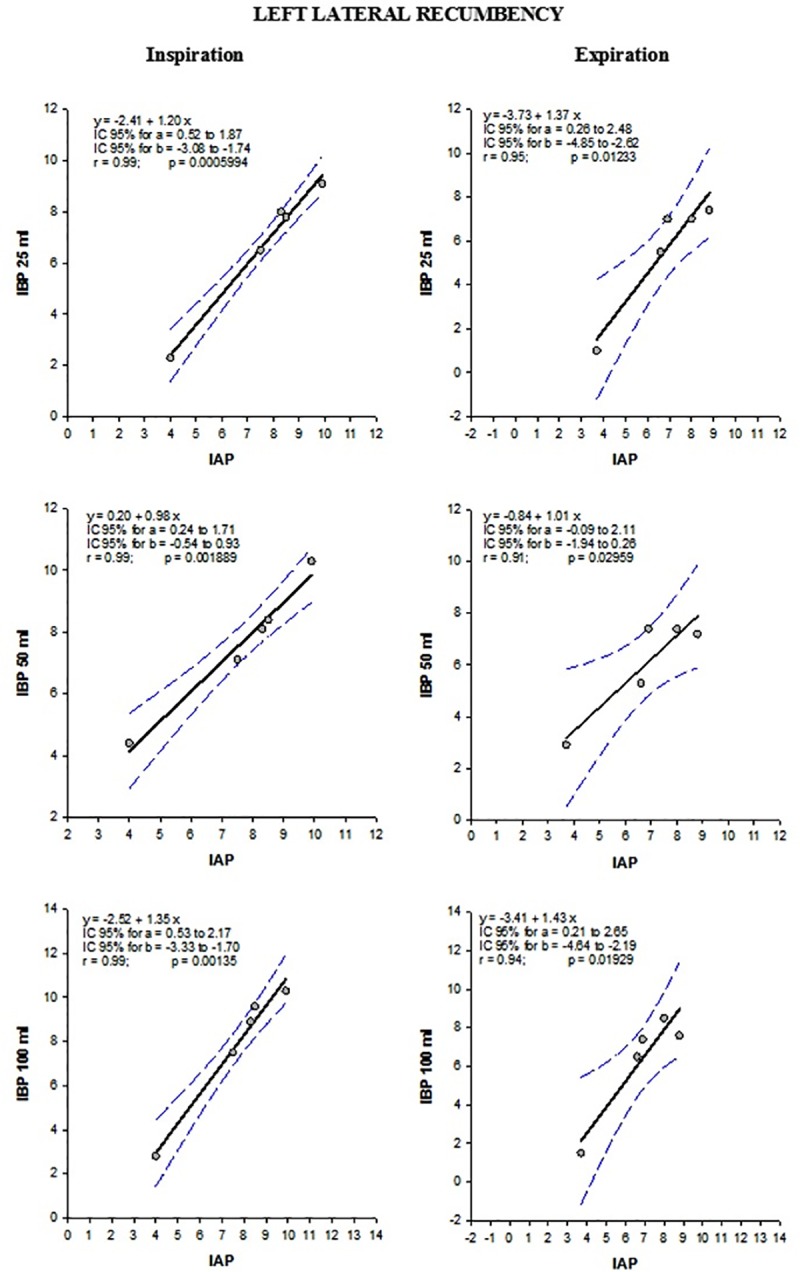
Ordinary least product (OLP) regression analysis of five equine patients in left lateral recumbency during elective surgical procedures. Ordinary least product (OLP) regression analysis between intra-abdominal pressure (IAP) and intrabladder pressure (IBP). Intrabladder pressure was obtained with the bladder distended with 25ml, 50ml and 100ml. Both pressures were recorded in five equine patients at end-inspiration and end-expiration whilst in left lateral recumbency during elective surgical procedures.

When results for left and right lateral recumbency were separated, a difference (p <0.05) between IAP and IBP_25ml_ was observed in patients in left lateral recumbency at end-inspiration and end-expiration (Table 6). Despite this difference, a very strong correlation was obtained between both pressures, regardless of the bladder distension volume. At end-inspiration, the coefficient of correlation (rs) between both pressures varied from 0.726 for the IBP_0ml_ to 0.931 for the IBP_50ml_. At end-expiration, the correlation coefficient (rs) between both pressures varied from 0.591 for the IBP_0ml_ to 0.937 for the IBP_25ml_ (p <0.001). Similarly, for this recumbency (left lateral), the Bland-Altman limits of agreement method showed greater agreement between IAP and IBP_50ml_ and IBP_100ml_ at end-inspiration (Table 7). The only distension volume for which there was no fixed and proportional bias by ordinary least products regression analysis (OLP) was 50 ml, regardless of the phase of the respiratory cycle. The other distension volumes had fixed bias between IAP and IBP, with no proportional bias (Table 4 and Fig 3).

**Table 6:** Intra-abdominal pressure and intrabladder pressure of five equine patients in left lateral recumbency during elective surgical procedures.

Patients	IAP		IBP							
	E. I.	E. E.	0 ml		25 ml		50 ml		100 ml	
			E. I.	E. E.	E. I.	E. E.	E. I.	E. E.	E. I.	E. E.
1	9.930	8.753	10.224	7.797	9.121	7.356	10.298	7.208	10.298	7.650
2	8.312	6.877	9.562	8.827	7.981	6.988	8.091	7.356	8.900	7.356
3	4.046	3.678	2.280	1.067	2.317	0.933	4.413	2.942	2.795	1.471
4	8.496	7.981	6.988	6.620	7.760	6.988	8.385	7.356	9.562	8.459
5	7.521	6.583	8.569	6.399	6.455	5.480	7.135	5.278	7.466	6.510
$\bar{X}$ ± SD	**8.31 ± 2.2**	**6.88 ± 1.9**	**8.57 ± 3.2**	**6.62 ± 3.0**	7.76 ± 2.6*	6.99 ± 2.7*	**8.09 ± 2.15**	**7.21 ± 1.9**	**8.90 ± 3.0**	**7.36 ± 2.8**

Pressure values expressed in mmHg.

* Statistically different from IAP (Dunnett's test, p ≤ 0.05).

Mean ($\bar{X}$) and standard deviation (SD) of intra-abdominal pressure (IAP) and intrabladder pressure (IBP) of five equine patients in left lateral recumbency during elective surgical procedures. Both pressures were recorded at end-inspiration (E.I.) and end-expiration (E.E.). Intrabladder pressure was recorded with the emptied bladder (0ml) and distended with predetermined volumes of 25ml, 50ml and 100ml.

**Table 7:** Bland-Altman limits of agreement method between intra-abdominal pressure and intrabladder pressure of 10 equine patients maintained in right (n = 5) and left (n = 5) lateral recumbency during elective surgical procedures.

Distension Volume	L. R. Right						L. R. Left					
	E. I.			E. E.			E. I.			E. E.		
	B	S. D.	CI 95%	B	S. D.	CI 95%	B	S. D.	CI 95%	B	S. D.	CI 95%
**0 ml**	-1.9	2.3	-2.5 - -1.2	-1.2	1.8	-1.7 - -0.7	-0.1	1.4	-0.5–0.3	-0.6	1.6	-1.1 - -0.2
**25 ml**	-1.6	2.0	-2.2 - -1.1	-1.6	1.8	-2.1 - -1.1	-0.9	0.6	-1.1 - -0.8	-1.2	0.9	-1.5 - -0.9
**50 ml**	-1.8	2.2	-2.4 - -1.2	-1.6	2.1	-2.2 - -1.1	**0.0**	0.6	-0.2–0.2	-0.7	0.8	-1.0 - -0.5
**100 ml**	-0.4	2.7	-1.1–0.4	-0.3	2.4	-0.9–0.4	**0.1**	0.9	-0.1–0.4	-0.4	1.0	-0.7 - -0.2

Pressure values expressed in mmHg.

Bias (B), standard deviation (SD) and confidence interval (CI) between the mean values of intrabladder pressure (IBP) and intra-abdominal pressure (IAP), obtained by the Bland-Altman limits of agreement method. The IAP and IBP values were obtained at end-inspiration (E.I.) and end-expiration (E.E.), with patients (n = 10) in right lateral recumbency (L.R. Right) (n = 5) and left lateral recumbency (L.R. Left) (n = 5). Intrabladder pressure was recorded with the bladder emptied (0ml) and with the bladder distended with predetermined volumes of 25ml, 50ml and 100ml.

Bladder distension volume also influenced IBP values in left lateral recumbency. Intrabladder pressure recorded whilst the bladder was distended with 25 ml differed (p <0.05) from the other IBPs both at end-inspiration and end-expiration. In spite of this difference, a strong correlation was also observed between IBPs, regardless of whether the bladder was emptied or distended. At end-inspiration, the correlation coefficient (rs) varied (p <0.001) from 0.687 (IBP_0ml_ and IBP_50ml_) to 0.956 (IBP_50ml_ and IBP_100ml_). At end-expiration, the correlation was weaker but still highly significant (p <0.001). The coefficient of correlation (rs) varied from 0.572 (IBP_0ml_ and IBP_100ml_) to 0.822 (IBP_25ml_ and IBP_50ml_).

Intra-abdominal pressure and IBP_0ml_ also differed (p <0.05) in patients in right lateral recumbency, at end-inspiration (Table 8). At end-expiration, this difference was observed between IAP and both IBP_25ml_ and IBP_50ml_. Despite pressure differences, IBP_0ml_ moderately correlated with IAP (rs = 0.560, p <0.001) at end-inspiration. For the other distension volumes, correlation between pressures was more significant (p <0.001). The coefficient of correlation (rs) varied from 0.751 for the IBP_100ml_ to 0.857 for the IBP_25ml_.

**Table 8:** Intra-abdominal pressure and intrabladder pressure of five equine patients in right lateral recumbency during elective surgical procedures.

Patients	IAP		IBP							
	E. I.	E. E.	0 ml		25 ml		50 ml		100 ml	
			E. I.	E. E.	E. I.	E. E.	E. I.	E. E.	E. I.	E. E.
**6**	8.680	6.841	4.671	3.420	3.347	2.096	2.905	1.839	3.273	2.207
**7**	11.328	10.298	10.224	9.158	10.702	8.532	9.783	7.797	13.681	11.033
**8**	12.026	9.415	12.505	10.482	11.512	9.599	11.806	9.893	12.836	10.960
**9**	11.732	10.666	11.843	10.886	11.585	9.415	11.659	9.562	11.916	9.673
**10**	16.256	12.817	11.475	10.114	14.711	12.505	14.969	12.725	16.532	14.656
$\bar{X}$ **± SD**	**11.73 ± 2.7**	**10.30 ± 2.2**	11.48 ± 3.2*	**10.11 ± 3.1**	**11.51 ± 4.2**	9.42 ± 3.8*	**11.66 ± 4.5**	9.56 ± 4.1*	**12.84 ± 5.0**	**10.96 ± 4.6**

Pressure values expressed in mmHg.

* Statistically different from IAP (Dunnett's test, p ≤ 0.05).

Mean ($\bar{X}$) and standard deviation (SD) of intra-abdominal pressure (IAP) and intrabladder pressure (IBP) of five equine patients in right lateral recumbency during elective surgical procedures. Both pressures were recorded at end-inspiration (E.I.) and end-expiration (E.E.). Intrabladder pressure was recorded with the bladder emptied (0ml) and with the bladder distended with predetermined volumes of 25ml, 50ml and 100ml.

**Fig 4:**
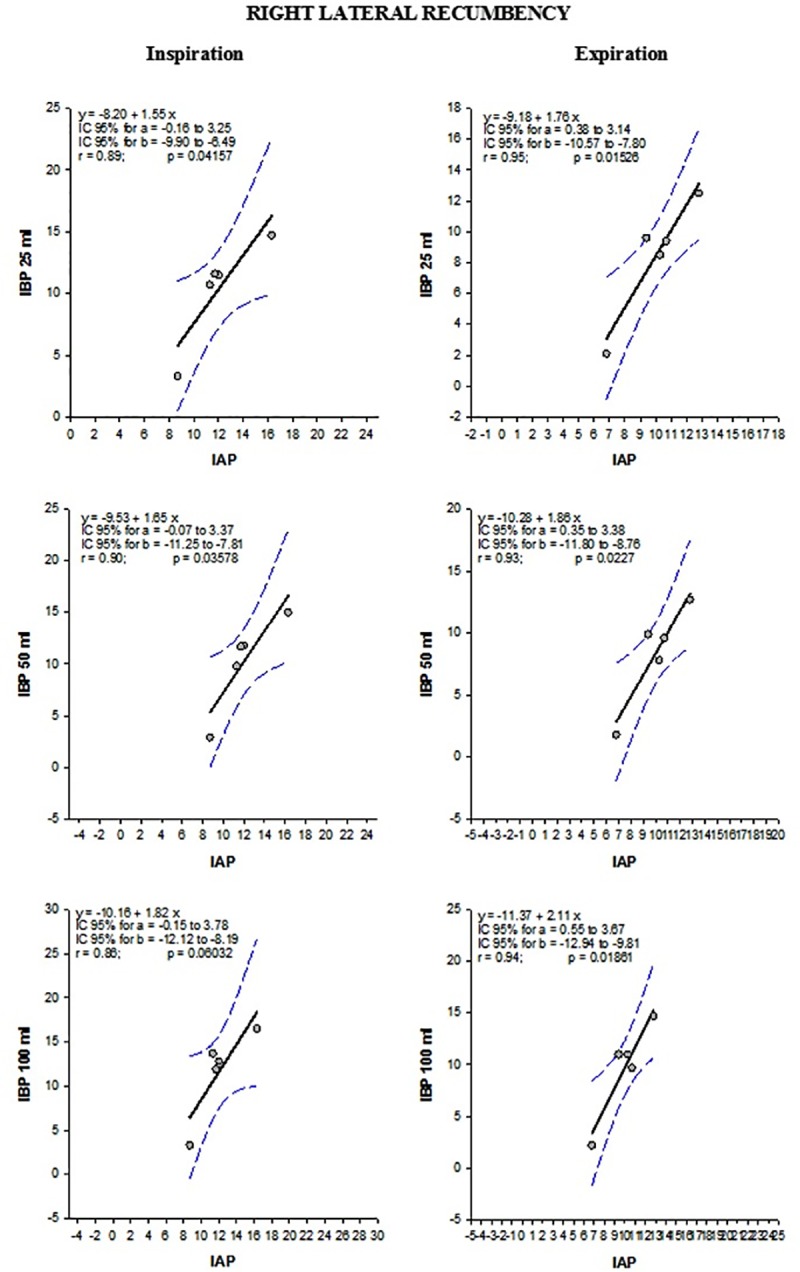
Ordinary least product (OLP) regression analysis of five equine patients in right lateral recumbency during elective surgical procedures. Ordinary least product (OLP) regression analysis between intra-abdominal pressure (IAP) and intrabladder pressure (IBP). Intrabladder pressure was obtained with the bladder distended with 25ml, 50ml and 100ml. Both pressures were recorded at end-inspiration and end-expiration in five equine patients kept in right lateral recumbency during elective surgical procedures.

For patients in right lateral recumbency, a moderate correlation was observed between IAP and IBP_0ml_ (rs = 0.442, p <0.001) at end-expiration, and a strong correlation was observed between both pressures whilst the bladder was distended, regardless of the volume used. The correlation coefficient (rs) varied (p <0.001) from 0.668 for the IBP_50ml_ to 0.724 for the IBP_25ml_. For this recumbency (right lateral), the Bland-Altman limits of agreement method showed greater agreement between IAP and IBP_100ml_, independent of the phase of the respiratory cycle (Table 8). For patients in right lateral recumbency ordinary least product (OLP) regression analysis indicated a fixed bias but no proportional bias between IAP and IBP, for both an emptied and distended bladder at both end-inspiration and end-expiration (Table 4 and Fig 4).

Similarly to findings in other body positions, bladder distension volume influenced IBP values in patients in right lateral recumbency. Intrabladder pressure recorded whilst the bladder was distended with 100 ml differed (p <0.05) from the other IBPs at end-inspiration. At end-expiration, only IBP_25ml_ and IBP_50ml_ did not differ from one another (p> 0.05). There was a weak correlation between IBP_100ml_ and the other IBPs at both end-inspiration (rs> 0.206, p <0.001) and end-expiration (rs> 0.214, p <0.001). Conversely, there was a strong correlation between IBP_25ml_ and IBP_50ml_ at both end-inspiration (rs = 0.913, p <0.001) and end-expiration (rs = 0.949, p <0.001). There was a moderate correlation (p <0.001) between the IBP recorded with the emptied bladder and IBP_25ml_ at end-inspiration (rs = 0.582) and with IBP_50ml_ at end- expiration (rs = 0.602).

## Discussion

In contrast to previous descriptions [13,17,18,25], correlation and concordance between IAP and IBP were observed with patients in lateral recumbency, regardless of which side the animals were positioned on (right or left). This finding has not been previously reported in horses. The majority of the most recent IAP recordings in this species used the direct method of intra-abdominal pressure acquisition because there was a scientific rationale to support the notion that indirect methods could not be applied to equine species [13,18]. However, in all previous trials, measurement were made with animals in a standing position [13,17–19,25].

This study finds that body positioning is an important factor in evaluating efficacy of this technique, similar to previous findings in man [1]. However, in human patients, individuals must be positioned in the supine position for the estimation of IAP via IBP. In this position, the urinary bladder is positioned at the height of the median axial line, making the methodology viable [4,10]. However, in the animals evaluated, lateral recumbency was found to be more appropriate than the dorsal recumbency.

For both body positions, the calibration point of the saline column was fixed at the site estimated to be the height of the mean axial line of each animal. During evaluation in dorsally recumbent patients, the saline column was calibrated at the level of the ischial tuberosity [13,18]. In human patients in lateral recumbency, calibration was done at the level of the pubic symphysis [18,25].

There was a difference between the mean values of IAP in relation to the IBP as well as a correlation and strong agreement between both pressures. For the patients in lateral recumbency there was also correspondence between the two by the multivariate analysis, independent of the volume used for bladder distension. Therefore, despite the fact that the IBP (average) does not accurately mirror the IAP, for all volumes of distension it is possible to use IBP to monitor pressure variations that may occur within the abdominal cavity of horses.

The most significant correlations between the pressures were obtained when the IBP was recorded by distending the bladder with 50ml and 100ml, in both lateral recumbencies (right or left). These findings corroborate what has been recommended in man [4,10,11,14,15]. The use of small volumes for urinary bladder insufflation in horses prevents excessive distension such that contact of the bladder wall with intestinal loops is avoided, similar to recommendations in human medicine [4,10,11,14,15]. Thus, the compressive effect of the intestinal loops on the bladder wall, which would interfere with the IBP values in lateral recumbency, is minimized, irrespective to the side on which the patient is positioned.

The use of the ordinary least products regression analysis (OLP) to determine possible biases between IAP and IBP, has advantages over the traditional least square regression method. Mean bias, as determined by the Bland and Altman technique, is obtained through the interaction of fixed and proportional biases, so does not fully reflect the fixed bias of the methodologies being compared [26]. Moreover, using only the regression of the least squares to describe proportional bias level through the slope of the line can produce distorted results, since any error is attributed only to the dependent variable (y-axis). It assumes error is only introduced by the method that is being compared to the ‘gold standard’ methodology. The use of OLP to compare two methodologies avoids these two limitations, allowing precise determination of any fixed or proportional bias [23,24,26].

Comparison between IAP and IBP_50 ml_ in left lateral recumbency revealed no proportional bias at either end-inspiration and end-expiration, indicating satisfactory linearity between the pressures. Also, this distension volume was the only one that presented no fixed bias between pressures, regardless at which end of the respiratory cycle they were measured. Thus, for this distension volume and in this body position, the pressures were interchangeable with each other. Therefore, the results obtained using one method could be extrapolated to the other [26]. The other volumes of bladder distension showed satisfactory linearity (without proportional bias), but did have fixed bias between pressures. Thus, pressures could not be considered to be interchangeable. Consequently, the most suitable volume for bladder distension during indirect assessment of intra-abdominal pressure in anesthetized and mechanically-ventilated horses is 50 ml.

After catheterization of the urinary bladder, an adaptation period of five minutes was allowed, following previous recommendations for human subjects [4,22] and equine cases [13,18], in which a minimum adaptation period of two minutes was recommended to avoid the influence of detrusor muscle contraction on the values of the IBP [4,18,22].

Similarly, bladder distension with excessive volumes can also activate the detrusor muscle, directly interfering with the IBP values [12]. As a result of this in human patients it is recommended that the bladder should be distended with small volumes (25ml preferably) [4,10,22,27]. In horses, because the upper motor neurons also mediate urethral sphincter tone, the urinary bladder can accumulate large volumes of urine, with only a slight increase in intrabladder pressure [28].

The volumetric capacity of the urinary bladder in people ranges from 400 to 600ml. However, filling with volumes above 150ml is enough to trigger the micturition stimulus [29]. In horses, the urinary bladder can hold between 2.8 and 4.5L of urine [30]. When the efficacy of the indirect IAP estimation by IBP in standing female horses was tested, it was observed that the volumes of bladder distension used (976.57 ± 164.42 ml) would apparently have been sufficient to stimulate urination in the animals evaluated [13]. However, it has been reported that these animals had the capacity to retain volumes up to 4.0L without triggering the urination stimulus [31].

In human patients, the recommended volume of bladder distension of 25 ml for the indirect assessment of the IAP, corresponds to only 4% of the volumetric capacity of the urinary bladder (600 ml). In the animals evaluated, we obtained a close relationship between pressures when using a bladder distension of 50ml (1.3%). Therefore, the distension values used did not significantly influence the IBP values.

Hypothetically, the distension of the bladder with significant fluid volumes may overestimate the IBP values in relation to the IAP in both lateral recumbencies, as has been documented in horses in a standing position [13,18], and in human subjects [12]. Thus, the volume of bladder insufflation sufficient to cause excessive distension of the bladder wall and possibly wall compression by the intestinal loops or even detrusor muscle action [12,18,22] is still unknown in horses.

In our study, there was no confirmation of complete emptying of the bladder (either by rectal palpation or ultrasonographic evaluation) at the beginning of the study, or even between bladder distensions at predetermined volumes. There is no simple, direct and objective procedure to ensure the bladder has been completely emptied, even in human subjects [4]. One could as well consider the endoscopic approach for horses, but to use endoscopy just to ascertain that the bladder is completely emptied certainly lacks practicality. We believe that transrectal ultrasonography should be a more prompt and practical approach, especially for the laterally recumbent horse.

The daily urinary output of a horse can vary from three to seven liters, reaching a maximum of 10 liters in 24 hours [32]. A production of seven liters/day equates to approximately 4.8 mL/min. In that sense, even if the urinary bladder of the animals evaluated was not completely emptied, the residual volume of urine added to the amount produced during the evaluations, was certainly not enough to interfere with the IBP values obtained. Allowing for the time required to record the IBP and the five-minute adjustment period, the urinary bladder was emptied approximately every ten minutes. In this period, the volume of urine produced (48ml for a daily volume of 7.0L) would have been insufficient to promote detrusor muscle stimulation and therefore affect measured IBP values.

In addition to the distension volume, the visceral anatomy of horses and the gravitational and shear forces within the abdominal cavity may directly influence the IBP values, as it does on IAP values [6,12], in response to variations in body position, mainly when patients (horses) are in dorsal recumbency. This means it is important to avoid this body position during indirect measurement of IAP in horses. Anatomically, the urinary bladder of large animals is confined to the pelvic cavity, as in man [33]. In carnivores, the bladder is located in the pelvic cavity, but as pressures increase, it extends towards the abdomen [34].

Although there is no anatomical distinction between the location of the organ in the different species, it is believed that the equine colon, by virtue of its size and capacity of distension and storage, exerts pressure on the wall of the urinary bladder of horses in a dorsal recumbency and, consequently results in overestimation of the values of IBP in comparison to IAP. This may be the primary reason why the dorsal recumbency was found to be inappropriate for the indirect estimation of the IAP by the IBP in our patients.

There were considerable differences between measurements made in right and left lateral recumbencies. The highest correlation, concordance (association), and the absence of fixed and proportional bias between pressures were observed in patients in left lateral recumbency. In this position only a few IBP values differed statistically from the IAP. Fixation of the equine ascending colon is limited to the right dorsal colon, which is attached to the abdominal ceiling at the base of the cecum, to the mesenteric root and to the pancreas, allowing the left colon to rotate on its axis [34]. Hypothetically, this mobility, when the animal is in right lateral recumbency, could also exert a compressive force (with less intensity) on the wall of the urinary bladder. This fact, related to the anatomical characteristics of the equine colon, may have influenced the IBP values of the animals in right lateral recumbency, similar to animals in dorsal recumbency. However, based on the correlation between both pressures for this body position, the interference of the intestinal loops on the IBP values must be small when compared to dorsal recumbency, under our experimental conditions (water and food fasting and patients anesthetized and maintained under controlled mechanical ventilation). This fact would also explain the difference between the results obtained for both lateral recumbencies.

When considering the body position and phase of the respiratory cycle, the most significant correlations and concordances, with absence of fixed and proportional bias were obtained at either end-inspiration and end-expiration, with the patient in left lateral recumbency. These observations differ from the recommendations established for human patients, in which it is recommended that the recording of IBP values takes place at the end of expiration [1,4]. In spontaneous breathing, intrathoracic pressure is negative during inspiration, and this does not occur when positive pressure ventilation is used. In a ventilated subject, airway pressure remains positive throughout the cycle [21,35,36]. This could explain why it was possible to record the IBP at either end-inspiration and end-expiration in our patients, but not in man, since people are not usually ventilated during the procedure.

## Conclusion

There was no correlation between the IAP and IBP in equine patients whilst dorsally recumbent, regardless of the volume of bladder insufflation. Therefore, this position is not suitable for indirect measurement of IAP in horses. As well as the standard method for recording IAP in human patients, IBP can also be used in for monitoring IAP in horses. However, unlike in human medicine, equine patients should be positioned in left lateral recumbency, ideally using a 50 ml bladder filling volume. In mechanically-ventilated horses, IBP values can be recorded at the end of both phases of the respiratory cycle.
